# Why Do We Take Risks? Perception of the Situation and Risk Proneness Predict Domain-Specific Risk Taking

**DOI:** 10.3389/fpsyg.2021.562381

**Published:** 2021-03-08

**Authors:** Carla de-Juan-Ripoll, Irene Alice Chicchi Giglioli, Jose Llanes-Jurado, Javier Marín-Morales, Mariano Alcañiz

**Affiliations:** ^1^Institute for Research and Innovation in Bioengineering, Polytechnic University of Valencia, Valencia, Spain; ^2^Universitat Politècnica de València, Valencia, Spain

**Keywords:** risk taking, locus of control, emotion regulation, executive control, personality, sensation seeking impulsivity

## Abstract

Risk taking (RT) is a component of the decision-making process in situations that involve uncertainty and in which the probability of each outcome – rewards and/or negative consequences – is already known. The influence of cognitive and emotional processes in decision making may affect how risky situations are addressed. First, inaccurate assessments of situations may constitute a perceptual bias in decision making, which might influence RT. Second, there seems to be consensus that a proneness bias exists, known as risk proneness, which can be defined as the propensity to be attracted to potentially risky activities. In the present study, we take the approach that risk perception and risk proneness affect RT behaviours. The study hypothesises that locus of control, emotion regulation, and executive control act as perceptual biases in RT, and that personality, sensation seeking, and impulsivity traits act as proneness biases in RT. The results suggest that locus of control, emotion regulation and executive control influence certain domains of RT, while personality influences in all domains except the recreational, and sensation seeking and impulsivity are involved in all domains of RT. The results of the study constitute a foundation upon which to build in this research area and can contribute to the increased understanding of human behaviour in risky situations.

## Introduction

Risk taking (RT) is a component of the decision-making process in situations that involve uncertainty and in which the probability of all outcomes – rewards and/or negative consequences (Brand et al., 2007) – is already known (Bechara et al., 2005; Krain et al., 2006). Risk takers tend to make decisions with both high potential benefits and high potential adverse outcomes, rather than choosing more cautious alternatives (Slovic, 1987; Mellers et al., 1997). The decision-making process is influenced by three main elements: decision features, situational factors, and individual differences (Einhorn, 1970; Hunt et al., 1989). Decision features are the characteristics of the decision itself, such as the ordering of the choice options (Appelt et al., 2011) and situation framing (Levin et al., 2002). Situational factors refer to the context of the decision, for example, time pressure (Dror et al., 1999). Individual differences are the third main factor in the decision-making process. Appelt et al. (2011) argued that, although the influence of individual differences in decision making has been widely studied, there is no consensus as to how to interpret these relations.

Some authors have identified the perception of benefits, the perception of risks, and risk attitude – “how much risk they [the subjects] are willing to accept in exchange for a specific return” (Figner and Weber, 2011; p. 212) – as the individual factors that may drive RT. Within this framework, the influence of the cognitive and emotional processes in decision making may affect the way in which a risky situation is perceived; they have also been identified as key elements of individual differences that may affect RT. First, an inaccurate assessment of a situation may constitute a perceptual bias in decision making, which might influence RT. In situations in which “hot” affective processes are prominent (e.g., condom use; Figner and Weber, 2011), emotion regulation skills – the control of emotions (Gross, 2002) – and internal locus of control – the perception that events are under one’s own control (Rotter, 1966) – have been highlighted as influential factors in the “cooling process” (Crisp and Barber, 1995; Miu and Crişan, 2011). In addition, executive control is the ability to control thoughts to inhibit or adapt behaviours according to the situation (Diamond, 2013). It involves top-down mental processes that require the individual to make an effort, meaning that the process is not automatic. Individuals with low executive control have been shown to more poorly evaluate situations and search for less information before making decisions, which can lead to risky behaviours (Magar et al., 2008). Finally, there seems to be consensus across different domains that risk proneness influences RT. This trait has been defined as the propensity to be attracted to potentially risky activities (Raffaelli and Crockett, 2003), and could be considered a cross-situational trait in RT as it has been related to temperamental aspects, such as sensation seeking and impulsivity (Zuckerman and Kuhlman, 2000). Indeed, while some individuals are characterised by strong directional risk proneness, others are situation-sensitive (Weber and Milliman, 1997; Nicholson et al., 2002; Weber et al., 2002). In the latter cases, the decision-making process may be highly dependent on decision features and situational factors. In light of these results, we consider it necessary to study these findings in an aggregated way, and provide clear conclusions regarding the influence of perceptual and cognitive biases in RT. In the following sections, the psychological dimensions that influence RT both in perceptual processes and risk proneness are discussed and the aim of our study is presented.

### Individual Differences in the Perception of Benefits and Risks

#### Locus of Control

Rotter (1966) found that locus of control indicates the degree to which an individual perceives events to be under his/her control (internal control) or under the control of outside forces, such as fate or other people (external control). Marsh and Richards (1986) identified five factors for the Rotter’s locus of control scale: general luck, which is related to attributing one’s life course to luck or chance; political control, which refers to low expectations of influencing political institutions and world affairs; personal initiative, which attributes to the influence of external elements in their work and personal situation rather than to the effort of oneself; interpersonal control, which refers to the little control of one’s influence over other people; and academic situation, which is related to the attribution to the influence of external elements in their academic results. The relation between locus of control and RT has been widely examined, although it seems that previous studies have reached opposite conclusions, based on the nature of the situations examined. Individuals with an internal locus of control have been shown to take more risks in some areas, such as the civil rights struggle (Gore and Rotter, 1963), the military (Higbee, 1972) and in entrepreneurship (Ahmed, 1985). Conversely, other studies have found that individuals with an internal locus of control take less risks in the domains of forestry and construction (Salminen and Klen, 1994), sexual practices (Terry et al., 1993) and piloting (You et al., 2013). Crisp and Barber (1995) suggested that individuals with an internal locus of control more accurately assess situations. Thus, locus of control may influence how situations are perceived, but not necessarily RT. Instead, it might be expected that internals, who perceive greater risk, would make safer decisions. In contrast, externals may perceive situations as if they are under other people’s control.

#### Emotion Regulation

Emotion regulation is the control of emotions (Gross, 2002). It can influence three components of RT, which involve different deliberative-versus-automatic strategies: interrupting a risk behaviour, thinking before acting, and choosing between two alternatives (Steinberg, 2004). Emotion regulation can be applied through two strategies, cognitive reappraisal and expressive suppression. Cognitive reappraisal is an antecedent-focused strategy that involves changing the meaning of a situation by reformulating the way it is understood to minimize or modify its emotional impact (Gross and John, 2003). It allows individuals to psychologically distance themselves from situations (Mischel and Ayduk, 2004). In contrast, the response-focused strategy of expressive suppression is the inhibition of the emotional response associated with a particular emotion (Gross and John, 2003). Generally, suppression is understood to be a maladaptive strategy, which involves an active effort sustained over time, while reappraisal is considered to be an adaptive strategy that modifies the emotion at an early stage (Gross, 2002; Evers et al., 2010). The relation between the habitual use of either emotion-regulation strategy and RT does not appear to be entirely established. Some studies have suggested that individuals who use cognitive reappraisal tend to take greater risks, as this strategy mitigates the influence of negative emotions, which leads them to be less sensitive to both the probability and the magnitude of potential losses (Heilman et al., 2010; Panno et al., 2013). On the other hand, some authors have suggested that reappraisal is related to positive affect and lower RT, in domains such as smoking, risky drinking (Magar et al., 2008; Fucito et al., 2010) and emotional eating (Evers et al., 2010). These results suggest that the relation between emotion regulation strategies and RT relies heavily upon the decision-making context. Hence, we may find positive relations between reappraisal strategy and RT in the contexts in which the positive outcomes are perceived as more salient than the negative consequences, or in which RT is not necessarily considered to be a maladaptive behaviour (Duell and Steinberg, 2019; Pellegrino, 2019), such as in entrepreneurship or social situations. In contrast, emotional suppression strategies may be positively related to RT in contexts in which the negative outcomes are perceived as more salient than the positive outcomes, or in which RT is clearly a maladaptive behaviour, such as health and ethical RT (Duell and Steinberg, 2019; Pellegrino, 2019).

#### Executive Control

Executive control has an important role in decision making (Eslinger and Damasio, 1985; Manes et al., 2002; Del Missier et al., 2010) as it operates in perception, conflict resolution, and retention processes (Pessoa, 2009). The relation between executive control and RT has been widely examined in adolescents and young adults, as these groups tend to show less cognitive control, particularly when facing situations with desirable or immediately accessible rewards (Falk and Rickardsson, unpublished). These studies suggested that executive control, as a fundamental mediator in the inhibition of pleasurable stimuli, and in the development of adaptive behaviour patterns, might contribute to RT in some domains when it is weak, such as drug addiction (Kalivas and Volkow, 2005), prompting riskier behaviours in daily life (Pharo et al., 2011). Executive control is comprised of inhibition, working memory and cognitive flexibility (Diamond, 2013). Cognitive flexibility is the ability to adjust perspectives to adapt to the changing demands of a situation. It is related to the other two executive functions, since it requires inhibition to deactivate the previous perspective and working memory to activate a new perspective (Diamond, 2013). Deficits in cognitive flexibility have been shown to influence RT, leading to violent and offending behaviours (Vilà-Balló et al., 2015) as well as eating disorders (Perpiñá et al., 2017).

### Individual Differences in Risk Proneness

#### Personality: The Big Five-Factor Model

Personality has been found to have a strong influence on RT behaviours (e.g., Zuckerman and Kuhlman, 2000; De Vries et al., 2009). Individual personality trait differences influence risk proneness, as they involve motivational forces that promote risky decisions, insulation against concerns about negative consequences, and they act as cognitive barriers (Nicholson et al., 2002). Among the numerous personality models developed in psychology research, the Big Five-factor model of personality – composed of neuroticism, extraversion, openness, agreeableness and conscientiousness factors (McCrae and Costa, 1997) – seems to be the most generally recognized in the study of the relation between personality and risk behaviour. Neuroticism has been related to negative affect and sensitivity to punishment (Elliot and Thrash, 2010). High levels of neuroticism may lead to risk aversion in most domains, as a way of avoiding guilt or anxiety regarding negative outcomes. In contrast, there seems to be an inverse relation between neuroticism and RT in the health domain (Nicholson et al., 2005). In these cases, some studies identified a tendency to take risks to alleviate anxiety and other emotions (Vollrath and Torgersen, 2002). Nicholson et al. (2005) suggested that health-related RT is most strongly influenced by environmental factors, and least under the control of individual psychological disposition. Conversely, extraversion, as a generalized need for stimulation, is manifested in positive affect and sensitivity to reward (Eysenck, 1973), prompting RT behaviours (Lauriola and Levin, 2001). Openness to experience relates to cognitive risk seeking, acceptance of experimentation, and tolerance of uncertainty, change, and innovation (McCrae and Costa, 1997). Agreeableness, which is characterized by trust, straightforwardness, and compliance, has been related to risk aversion (Gullone and Moore, 2000; Hoyle et al., 2000). Conscientiousness, which is a need for compliance under conditions of conformity and control, has been related to risk avoidance (Nicholson et al., 2005; Schwebel et al., 2006).

#### Personality: Sensation Seeking

Sensation seeking has been defined as “the seeking of varied, novel, complex and intense sensations and experiences, and the willingness to take physical, social, legal, and financial risks for the sake of such experience” (Zuckerman, 1994; p. 27). Individuals with varying levels of sensation seeking may exhibit differences in arousal and attention, which leads to differential information processing (Zuckerman, 1979; Zuckerman and Como, 1983). Several studies have shown a positive relation between sensation seeking and RT in different domains, such as substance abuse, risky sexual behaviour, reckless driving, and vandalism (e.g., Donohew et al., 2000; Wagner, 2001). Zuckerman (1994) identified four dimensions of the sensation-seeking trait: thrill and adventure seeking, experience seeking, disinhibition, and boredom susceptibility. The thrill and adventure seeking dimension reflects a desire to engage in physical activities and is positively related to risky behaviours in driving and sports (Zuckerman, 1994; Wishart et al., 2017). The experience-seeking subtrait has been shown to be a predictor of the openness personality trait, due to its relation to arousal seeking through the mind and senses (Zuckerman, 1984; Roberti, 2004). High experience-seeking individuals present lower sensitivity to aversive stimulation (Netter et al., 1996), and tend to display risky substance use behaviours (Pedersen et al., 1989). Disinhibition is a significant predictor of RT in several domains, including rule-breaking behaviours and violations of societal norms (Donohew et al., 2000; Roberti, 2004; De Vries et al., 2009). Boredom susceptibility, which is intolerance for routine and repetitive activities (Zuckerman, 2006), tends to be reflected in RT behaviours in domains such as sports (Guszkowska and Bołdak, 2010).

#### Personality: Impulsivity

Impulsivity is defined as the “predisposition toward rapid, unplanned reactions to internal or external stimuli without regard to the negative consequences of these reactions to the impulsive individual or to others” (Moeller et al., 2001; p. 1784). Whiteside and Lynam (2001) argued that impulsivity is comprised of a set of five impulse-related traits: negative urgency, lack of premeditation, lack of perseverance, sensation seeking, and positive urgency. According to Whiteside and Lynam (2001) and Whiteside et al. (2005), negative and positive urgency traits relate to the tendency to exhibit impulsive behaviours when facing negative/positive situations. Lack of premeditation relates to thoughtless behaviours and to the tendency to favour alternatives with short-term rewards over options that might lead to more valuable but delayed rewards. Lack of perseverance reflects an absence of focus on a tedious or difficult activity. Sensation seeking is an attraction toward exciting, new, and potentially dangerous experiences.

Impulsivity has emerged as one of the strongest predictors of RT in different domains. Moreno et al. (2012) found that recreational cannabis consumption was associated with high levels of impulsivity and sensation seeking, and with inhibitory control deficits. Donohew et al. (2000) showed that impulsivity and sensation seeking were strongly related to some sexual RT indicators: intention to have sex, number of lifetime sexual partners, being pregnant or having caused a pregnancy, having unwanted sex when drunk, having unwanted sex under pressure, and using alcohol or having a partner who used alcohol before sex. Furthermore, this relation has been demonstrated in other contexts, such as gambling (Blanco et al., 2009) and alcohol use (Coskunpinar et al., 2013).

### The Current Study

**FIGURE 1:**
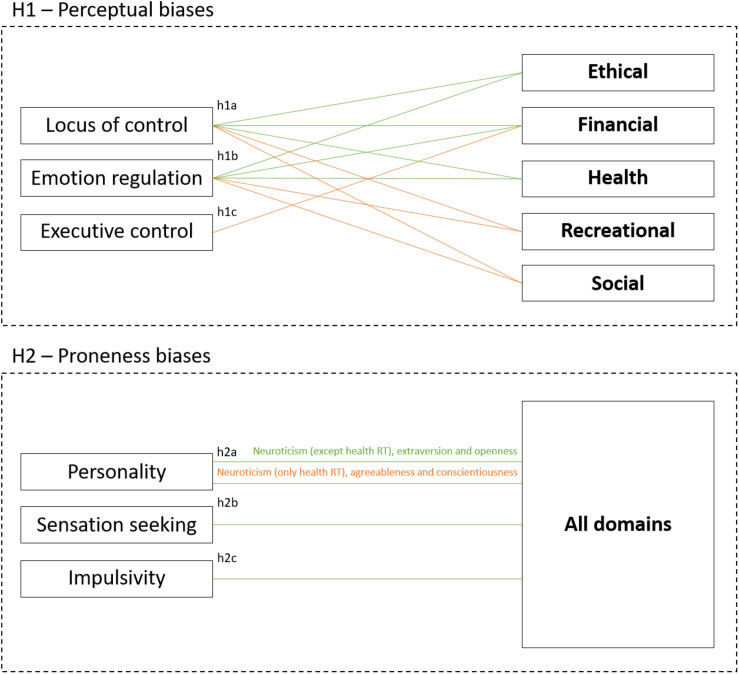
Study hypotheses. Red lines represent positive relation and orange lines represent negative relation.

The aim of the present study is to examine the relation between RT biases and risk behaviours, in order to identify the components of the cross-situational factors that influence RT and the variables that operate only in specific domains. This study aims to fill an existing gap in the literature, since there is no study, to our knowledge, that analyses the influence of psychological biases on RT from both, domain-dependent and cross-domain RT perspectives. The study hypotheses are the following (see Figure 1):

Hypothesis 1. Perceptual biases in RT: locus of control (h1a), emotion regulation (h1b), and executive control (h1c) are variables in the perception of benefits and risks in the decision-making process that each influence RT in those specific domains, requiring an accurate assessment of risks and benefits. On one hand, an internal locus of control and the use of the cognitive reappraisal strategy could lead to safe behaviours in the ethical and health domains. Additionally, financial decisions tend to involve complex situations, which require effortful processing – executive functions (Diamond, 2013) – to perceive and interpret each option. In this domain, high executive control would also be related to risk avoidance. On the other hand, recreational and social RT involve more salient potential positive outcomes, an internal locus of control, and the use of the cognitive reappraisal strategy which could lead to risky behaviours.

Hypothesis 2. Proneness biases in RT: personality (h2a), sensation seeking (h2b), and impulsivity (h2c) will influence RT consistently in all domains, constituting a trend toward risk proneness or risk avoidance, regardless of the type of risk. Regarding personality, neuroticism is expected to show a positive relation with RT in all domains, except in the case of health, in which it is expected to show a negative relation. Extraversion and openness are expected to appear as facilitators of RT, while agreeableness and conscientiousness may be related to safe behaviours. Sensation seeking and impulsivity are expected to show a positive relation with RT in all domains.

## Materials and Methods

### Participants

A total of 98 subjects balanced in terms of gender (50 men and 48 women) and age (35% under 30, 35% among 30–45, 30% above 45; mean age = 37.08, SD = 10.91) were recruited by a sampling company to participate in the experiment. The sample company contacted each participant and made an appointment for them to come to the laboratory. Before beginning the experiment, the participants gave their informed consent for their involvement. The responses were anonymised and randomised to ensure the privacy of the information. The study obtained prior ethical approval of the Ethical Committee of the Polytechnic University of Valencia.

### Measures

The risk-related constructs were assessed by means of a battery of self-reported measures and neuropsychological tests, which included the following:

Locus of control: Spanish version of the 23-item Rotter’s I-E scale (Rotter, 1966; Tous, 1984; Ferrando et al., 2011). This includes subscales for general luck, political control, personal initiative, interpersonal control, academic situation, and a total external locus of control score. The internal consistency of the scale in the present study was 0.613.

Emotion regulation: Spanish version of the 10-item Emotion Regulation Questionnaire (ERQ), which measures suppression and reappraisal strategies (Gross and John, 2003; Cabello et al., 2013). The Cronbach’s alpha coefficients previously reported for a Spanish sample were 0.75 for suppression and 0.79 for reappraisal (Cabello et al., 2013). The internal consistency of the scales in the present study was 0.77 for suppression and 0.73 for reappraisal.

Executive control: Two neuropsychological tasks were performed: Wisconsin Card Sorting Test (WCST; Grant and Berg, 1993), a measure of cognitive flexibility; and the Trail Making Task (TMT), a paper-and-pencil-based measure of attention and set switching (Reitan, 1958). To measure cognitive flexibility, we calculated the perseverative errors in the WCST. To assess attention and set switching, we measured the resolution times of parts A and B, respectively.

Personality: Spanish version of the NEO five-factor inventory (NEO-FFI). This comprises 60 items and includes the following factors: neuroticism, extraversion, openness, conscientiousness, and agreeableness (Costa and McCrae, 1989; Cordero et al., 1999). The reliability coefficients’ Cronbach’s alpha values ranged from 0.75 to 0.83 in a Spanish sample (Cordero et al., 1999). The internal consistency of the scales in the present study was: neuroticism α = 0.77, extraversion α = 0.85, openness α = 0.79, agreeableness α = 0.75, and conscientiousness α = 0.84.

Sensation seeking: Spanish version of the 40-item Sensation Seeking Scale-V (SSS-V) (Zuckerman et al., 1964; Pérez and Torrubia, 1986). This includes subscales for thrill and adventure seeking, experience seeking, disinhibition and boredom susceptibility, and a total sensation seeking score. The reliability coefficients’ Cronbach’s alphas ranged between 0.67 and 0.81 in a Spanish sample (Pérez and Torrubia, 1986). The internal consistency of the scale in the present study was: thrill and adventure seeking α = 0.81, experience seeking α = 0.54, disinhibition α = 0.63; boredom susceptibility α = 0.53, total sensation seeking α = 0.78.

Impulsivity: Short Spanish version of the UPPS-P impulsive behaviour scale (Whiteside and Lynam, 2001; Cándido et al., 2012). Composed of 20 items, this measures five impulsivity traits: negative urgency, lack of premeditation, lack of perseverance, sensation seeking, and positive urgency. The Cronbach’s alpha coefficients ranged from 0.66 to 0.81 in a Spanish sample (Cándido et al., 2012). The internal consistency of the scales in the present study was: negative urgency α = 0.72, lack of premeditation α = 0.77, lack of perseverance α = 0.78, sensation seeking α = 0.79, and positive urgency α = 0.60.

Risk taking: Spanish version of the Domain-Specific Risk-Taking (DOSPERT-30) scale (Blais and Weber, 2006; Lozano et al., 2017). This is a measure of the tendency to engage in real-life risk-taking behaviours in different domains, and includes the ethical, financial, health, recreation, and social subscales. Sample items include “Revealing a friend’s secret to someone else” (Ethical), “Betting a day’s income at a high-stake poker game” (Financial), “Riding a motorcycle without a helmet” (Health/Safety), “Moving to a city far away from your extended family” (Social), and “Going whitewater rafting at high water in the spring” (Recreational). Higher scores indicate greater RT in the domain of the subscale. The Cronbach’s alpha coefficients ranged from.64 to.85 in a Spanish sample (Lozano et al., 2017). The internal consistency of the scales in the present study was: Ethical α = 0.65, Financial α = 0.81, Health α = 0.68, Recreation α = 0.82, and Social α = 0.67.

### Procedure

The participants undertook the self-report questionnaires and completed the neuropsychological tasks on a personal computer. The process, which took place in an experimental room and was supervised by a research assistant, lasted approximately 45 minutes.

### Data Analysis

First, a multivariate outlier detection test was performed using all the features’ Mahalanobis distance between subjects, and thereafter a Chi-square test was performed on the Mahalanobis distance distribution. The subjects belonging to the far ends of the distribution, which was fixed for a *p*-value < 0.01, were defined as outliers; four outliers were found. Pearson correlations between each pair of numerical variables were computed to evaluate linear dependency. A prior power correlation analysis was performed, resulting in, for a population of 94 subjects, a Pearson coefficient of 0.285 achieving a power above 80%. Therefore, we only considered as significant the correlations that had a *p*-value lower than 0.05 and a Pearson coefficient higher than 0.285 in absolute value. Finally, multilinear regressions were computed to observe which input variables related to locus of control, emotion regulation, executive control, personality, sensation seeking, and impulsivity, explained the RT output variables. To explore the statistical importance of each variable in the multilinear regression model, a feature selection algorithm was implemented. In particular, a backward feature elimination (Guyon et al., 2008) was implemented based on the statistical analysis of the coefficient of each feature. This procedure of iterative feature selection would not miss any hidden relation between input variables; at the same time, it reduces the number of features used and increases the interpretability of the model. All input variables were normalised and an initial multilinear regression, including all inputs, was computed. The feature with the highest *p*-value was removed from the initial inputs, which resulted in a new set of inputs for the following regression. The computation of the *p*-value of the inputs was based on the null hypothesis that all the linear coefficients of the regression were zero. Due to the fact that a multilinear regression model considered different hypotheses simultaneously a Bonferroni correction was applied to the initial confidence interval chosen. The algorithm continued iteratively until the model included a set of inputs with every *p*-value under 0.05. Therefore, the coefficients of the features used in the multilinear regression are statistically different from zero, so all features contribute in the model. Once the backward elimination found a model in which all the variables are significant, it was preselected. In addition, three different checks were performed for the regression: the mean of the residuals had to be equal or close to zero, as well as the linear correlation between the input variable, and the residuals and the distribution of the residuals had to follow a normal distribution. If the multilinear regression model overcame these checks, it was considered as the final model; if it did not, the backward elimination continued. We obtained the *p*-value, the error, and the adjusted coefficient of determination of the regression model. A model was obtained for each RT subscale.

## Results

### Descriptive Analysis

The final dataset included 94 subjects between 20 and 51 years (49 males, 45 females; mean age = 35.77, SD = 10.65). Table 1 shows the statistical values of the subscales. This table includes a column indicating if the distribution of the subscales is normal or not according to a *t*-test fixing the *p*-value sensitivity to 0.05. Not normal distributions would achieve lower values than this threshold. According to the normality of each subscale, the mean and the standard deviation for normal distributions is shown or, in the case of not normal subscales, the median and the IQR is reported.

**TABLE 1:** Descriptive analysis of all variables, organized by subscales.

Scale		Subscale	Mean/Median	Std./IQR	Distribution	Range
Perceptual biases	Locus of control	General luck	3.00	2.00	Not normal	[0–6]
		Political control	3.00	2.00	Not normal	[0–5]
		Personal initiative	3.00	2.00	Not normal	[0–5]
		Interpersonal control	3.00	1.00	Not normal	[0–4]
		Academic situations	3.00	1.00	Not normal	[0–3]
		Locus of control (overall score)	3.00	4.00	Not normal	[2–21]
	Emotion regulation	Cognitive reappraisal	30.00	7	Not normal	[12–40]
		Emotional suppression	13.74	5.17	Normal	[4–26]
	Executive control	TMT Time Part A (ms)	4,2147	16,062.5	Not normal	[22,113–113,500]
		TMT Time Part B (ms)	45,668	16,008.75	Not normal	[23,675–80,787]
		WCST Perseverative Errors	35.00	33.39	Not normal	[0–91]
Proneness biases	Personality	Neuroticism	20.63	6.99	Normal	[2–37]
		Extraversion	32.95	7.31	Normal	[11–48]
		Openness	31.97	6.55	Normal	[14–48]
		Agreeableness	31.41	6.08	Normal	[14–43]
		Conscientiousness	32.69	7.25	Normal	[15–48]
	Sensation seeking	Thrill and adventure seeking	4.00	2.00	Not normal	[0–9]
		Experience seeking	7.00	2.00	Not normal	[3–10]
		Disinhibition	5.00	3.00	Not normal	[0–10]
		Boredom susceptibility	8.00	5.00	Not normal	[0–10]
		Sensation seeking (overall score)	23.09	5.71	Normal	[9–36]
	Impulsivity	Negative urgency	9.23	2.46	Normal	[4–16]
		Lack of premeditation	7.50	3.00	Not normal	[4–12]
		Lack of perseverance	7.00	3.75	Not normal	[4–14]
		Sensation seeking	10.26	2.51	Normal	[4–16]
		Positive urgency	10.00	2.00	Not normal	[5–14]
Risk taking		Ethical	14.00	8.00	Not normal	[6–28]
		Financial	17.00	9.75	Not normal	[6–42]
		Health	18.00	8.00	Not normal	[7–38]
		Recreational	26.50	15.50	Not normal	[7–42]
		Social	31.43	5.46	Normal	[18–42]

### Relation Between RT and the Risk-Related Constructs

**FIGURE 2:**
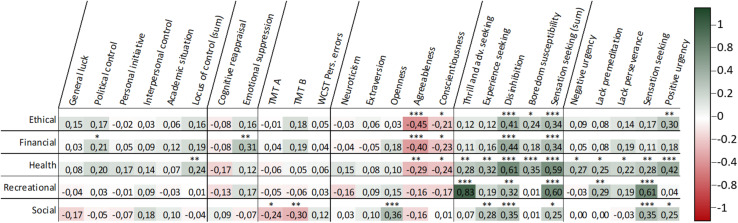
Correlation matrix obtained by Pearson coefficients between every pair of variables and the range of statistical significance by correlation. ^∗^*p* < 0.05, ^∗∗^*p* < 0.01, and ^∗∗∗^*p* < 0.001. Pearson coefficient of 0.285 achieves a power above 80%.

Figure 2 shows the Pearson Correlation coefficient between the RT scale and the variables considered as risk-related constructs.

After the statistical test, multilinear regressions were calculated to identify the most influential variables of the RT subscales. Table 2 lists the coefficient of each variable, including the weight and type of linear dependence (positive or negative).

**TABLE 2:** Statistical table showing the multilinear regressions for all output variables.

Predicted Variable	Bias	Input variable Risk taking	Input variable Risk avoidance	Coefficient	Model error	Adjusted *R* square
Ethical	Perceptual	-	Set switching (-TMT B)	−0.0001**	4.63	0.32***
	Proneness	-	Agreeableness	−0.3598***		
		Disinhibition	-	0.7050**		
Financial	Perceptual	-	Set switching (-TMT B)	−0.0001**	6.07	0.31***
	Proneness	-	Agreeableness	−0.3903***		
		Disinhibition	-	1.0920***		
Health	Proneness	Disinhibition	-	1.5342***	4.77	0.45***
		Lack of perseverance	-	0.6193**		
		Positive urgency	-	0.6669*		
Recreational	Proneness	Thrill and adventure seeking	-	2.2083***	4.75	0.72***
		Sensation seeking (UPPS-P)	-	0.8324***		
Social	Proneness	Openness	-	0.2608***	4.92	0.19***
		Disinhibition	-	0.6631**		

*p < 0.05, **p < 0.01, and ***p < 0.001.TMT B, Trail Making Task – Time Part B.

The first model, composed of set switching, agreeableness, and disinhibition, predicted 32% of the variance (*p* < 0.001, model error 4.63) of ethical RT. According to these results, ethical RT is predicted by both perceptual and proneness biases. The results showed that disinhibition promotes ethical RT, while set switching and agreeableness lead to ethical risk avoidance.

The second model, also composed of set switching, agreeableness, and disinhibition, predicted 31% of the variance (*p* < 0.001, model error 6.07) of financial RT. Financial RT is predicted by both perceptual and proneness biases. The results showed that disinhibition promotes financial RT, while set switching and agreeableness lead to financial risk avoidance.

The third model, composed of disinhibition, lack of perseverance, and positive urgency, predicted 45% of the variance (*p* < 0.001, model error 4.77) of health RT. Health RT is predicted only by proneness biases. The results showed that disinhibition, lack of perseverance, and positive urgency promote health RT.

The fourth model, composed of thrill and adventure seeking and sensation seeking, predicted 72% of the variance (*p* < 0.001, model error 4.75) of recreational RT. Recreational RT is predicted only by proneness bias. The results showed that thrill and adventure seeking and sensation seeking (impulsivity subtrait) promote recreational RT.

The fifth model, composed of openness and disinhibition, predicted 19% of the variance (*p* < 0.001, model error 4.92) of social RT. Social RT is predicted only by proneness biases. The results showed that openness and disinhibition promote social RT.

## Discussion

Risk taking is a component of the decision-making process in situations involving uncertainty and in which the probability of each outcome – rewards and/or negative consequences (Brand et al., 2007) – is previously known (Bechara et al., 2005; Krain et al., 2006). Risk takers tend to make decisions with both high potential benefits and high potential adverse outcomes, which can depend on perceptual and proneness biases. The results of this study provide a clearer view of the factors that affect RT, considering that some of them have a cross-domain influence, while the influence of others varies depending on the area or type of decision. This study aimed to fill this gap in the literature and expand this line of research in order to better understand decision-making processes in the face of risk. This study hypothesised that locus of control, emotion regulation, and executive control factors act as perceptual biases in RT, and that personality, sensation seeking, and impulsivity traits act as proneness biases in RT. The results are discussed below regarding the relation between RT in the various domains and the variables considered, as well as study limitations.

### Relation Between RT in the Different Domains and the Variables Considered

#### Perceptual Biases

First, we found moderate positive, significant correlations between emotional suppression and financial RT. Second, we found weak/moderate positive, significant correlations between set switching and social RT.

Regarding regression results, attentional control and set switching appeared as significant predictors of ethical and financial RT. Kim-Spoon et al. (2015) found that attentional control is a regulator of negative affect, which reduces the effects of anger and increases the effects of fear. These results suggest that, when subjects face situations in which they feel negative affect, high attentional control may lead to safe behaviours, for fear of the potential negative outcomes. Situations such as “Not returning a wallet you found that contains $200 – an item for ethical RT – or “Betting a day’s income on the outcome of a sporting event” – an item for financial RT – might generate the fear of damaging someone, being discovered, or even losing a large amount of money.

Hypothesis 1 posited that individuals with an external locus of control (h1a) and low emotional (h1b) and executive abilities (h1c), would show risky behaviours in those specific domains which require an accurate assessment of risks and benefits. First, we did not find significant relations between locus of control and RT, rejecting hypothesis 1a.

Second, the results showed that a relation exists between emotional suppression and financial RT, and not with the cognitive reappraisal strategy, which partially supports hypothesis 1b. The emotional suppression strategy is response-focused, modifying the behavioural aspect of the emotional response, but not the experience of negative emotions (Gross and John, 2003). Individuals tending to emotional suppression put things into perspective less frequently (Pellegrino, 2019) and require a cognitive effort to manage negative emotions (Gross and John, 2003). The use of the emotional suppression strategy might affect financial decision making, since it requires effortful processing to make decisions. The results for executive control suggested that attentional control and set switching lead to social RT and risk avoidance in the ethical and financial domains, partially supporting hypothesis 1c. RT can be classified as negative – illegal or dangerous – or positive – socially acceptable and constructive (Duell and Steinberg, 2019). The latter can be considered risky due to the variability and uncertainty of its potential consequences (Figueredo and Jacobs, 2010). Therefore, executive control seems to constitute a perceptual bias that drives positive RT, and to risk avoidance in domains in which taking risks involves potential negative outcomes. In the framework of social RT, Lahat et al. (2012) found that set switching ability in childhood allows knowing and considering both the positive and negative consequences of a situation, moderating the relationship between temperamental aspects and antisocial risk behaviours. In this domain, we could understand that executive control allows a more accurate analysis of the situation, perhaps avoiding social desirability biases that can modify the responses to situations presented as social RT on the DOSPERT scale, such as “Admitting that your tastes are different from those of a friend” or “Speaking your mind about an unpopular issue in a meeting at work.” Regarding the ethical and financial domains, executive control appears as a significant predictor of moral judgements and of gambling tasks, such that individuals with greater executive control show greater consistency in their responses (Moore et al., 2008; Blair et al., 2018). These results may suggest that greater consistency in the responses, mediated by executive control, indicates an adaptive RT derived from an accurate assessment of each situation.

#### Proneness Biases

First, the results showed moderate positive, significant correlations between openness and social RT. Agreeableness showed moderate/weak negative, significant correlations with RT in the ethical, financial, and health domains. Second, we found strong positive, significant correlations between thrill and adventure seeking and recreational RT. Furthermore, the results showed moderate positive, significant correlations between the experience seeking subtrait and health RT. In addition, disinhibition showed moderate/strong positive, significant correlations in all domains. Boredom susceptibility showed a weak/moderate positive, significant correlated with health RT. Third, the five impulsivity subtraits showed weak/moderate positive, significant correlations with health RT. Lack of premeditation also presented a weak positive, significant correlation with recreational RT, and sensation seeking presented a strong positive, significant correlation with recreational RT, and a weak positive, significant correlation with social RT. Finally, we found moderate positive, significant correlations between positive urgency and ethical RT.

Regarding regression results, the openness personality subtrait appeared as a significant predictor of social RT. The openness subtrait is relevant to an understanding of social attitudes, career changes, and moral reasoning (McCrae and Costa, 1997). The positive relation shown between social RT and openness is consistent with other studies (Josef et al., 2016) and this dimension of personality has been identified as a protector against social anxiety (Kaplan et al., 2015). Agreeableness, which is related to needs for compliance and control, was a significant predictor of ethical and financial risk avoidance, which is consistent with the results obtained by other authors (Nicholson et al., 2005; Soane et al., 2010).

The thrill and adventure seeking subtrait, which relates to the desire to engage in risky physical activities (Zuckerman, 1994; Wishart et al., 2017), appeared as a significant predictor of recreational RT. The recreational domain involves risky physical activities and dangerous situations, such as “Bungee jumping off a tall bridge.” This relation is consistent with other studies that found positive relations between the thrill and adventure seeking subtrait and risky driving and sport behaviours (Zuckerman, 1994; Wishart et al., 2017). Disinhibition, defined as a rule-breaking tendency (Donohew et al., 2000), appeared as a RT predictor in the ethical, financial, health, and social domains. Disinhibition could act as a RT facilitator in the ethical domain, inciting individuals to ignore previously established ethical norms. This result is consistent with other works that also found that the disinhibition subtrait is a significant predictor of ethical RT, specifically in academically dishonest behaviours (Weber et al., 2002; Etter et al., 2006). The influence of disinhibition on financial RT has been shown in different contexts, including gambling, in which it has a positive influence on frequency of expected future gambling (Wolfgang, 1988) and, recently, problem poker gambling, in which it is associated with the male gender and depression (Bonnaire and Barrault, 2018). The relation between disinhibition and health RT is well established, and has been demonstrated in different circumstances, such as substance abuse (Kopstein et al., 2001), alcohol consumption (Hittner and Swickert, 2006), and risky sex (Bancroft et al., 2003). Lastly, the influence of disinhibition on social RT has been confirmed by numerous studies, including those in which participants with high disinhibition scores showed high levels of violations of societal norms (De Vries et al., 2009) or social RT and expected benefits (Lozano et al., 2017).

Regarding impulsivity subtraits, lack of perseverance, which reflects an absence of focus on a boring or difficult activity, and positive urgency, which arises when an individual displays impulsive behaviours in positive situations (Whiteside and Lynam, 2001; Whiteside et al., 2005), were significant predictors of health RT. These results are also consistent with those obtained in other works, in which health RT was related to high scores in these impulsivity subtraits (e.g., Coskunpinar et al., 2013; Lozano et al., 2017). Situations such as “Engaging in unprotected sex” or “Sunbathing without sunscreen,” which are DOSPERT-30 scale items for health RT, involve salient positive rewards, which could explain this result. Lastly, sensation seeking (impulsivity subtrait) appeared as a significant predictor of recreational RT. The sensation seeking subtrait is defined as the attraction to exciting new and potentially dangerous experiences (Whiteside and Lynam, 2001; Whiteside et al., 2005) and has been related to recreational RT by other authors in activities such as high-risk sports (Gomà-i-Freixanet et al., 2012; Woodman et al., 2013).

In hypothesis 2, personality traits (h2a), sensation seeking (h2b), and impulsivity (h2c) were expected to have an influence on all RT, constituting a trend toward risk proneness or risk avoidance, regardless of the type of risk. First, the hypothesised relation between RT and openness, agreeableness, and conscientiousness was supported, partially accepting hypothesis 2a. Personality had an influence in all domains, except recreational. The results suggested that personality traits, in isolation, do not have an effect in all RT domains; however, personality, as the conjunction of personality traits, affects RT behaviours in almost all the domains studied. Second, our results suggested that sensation seeking is a bias toward risk proneness in various domains. Specifically, disinhibition was found to be a cross-domain subtrait that influences RT regardless of context, which supports hypothesis 2b. Third, we found relations between impulsivity subtraits and all RT domains. These results seem to suggest that impulsivity, which is involved in all domains of RT, has a traversal influence on risky behaviours, generating a general trend towards risk (RT or risk avoidance) regardless of the domain, supporting hypothesis 2c.

### Limitations

We acknowledge that the present study has some methodological limitations. First, to increase the statistical power of the analyses, the sample size could be larger. Second, the use of a single measure of RT may lead to biased results. As discussed previously, the scale might not encompass all the situations in which RT can be studied. In future studies, we intend to employ additional RT measures to complement the DOSPERT-30 scale, such as the Balloon Analogue Risk Task (BART; Lejuez et al., 2002), or the Bechara Gambling Task (Bechara et al., 1994), which enable close examination of all the potentially influential variables that affect subjects’ responses. Self-reported indexes of engagement in risky behaviours in daily life over specific periods of time (e.g., marijuana consumption during the previous year) have been used in other studies (Lejuez et al., 2003), and could be included. Third, self-reported measures might involve intrinsic biases (de-Juan-Ripoll et al., 2018), since individuals’ cognitive and psychological states may be different when answering the questionnaires as opposed to when they face real situations (Kivikangas et al., 2011). In addition, specific self-report items might be open to different interpretations (Lanyon and Goodstein, 1997), and some questions require people to possess overt knowledge of their dispositions (Schmitt, 1994), which is not always possible. In our future research, we will examine different RT metrics to identify ways of improving measurements, and investigate the application of virtual reality technologies in RT assessment.

## Conclusion

Examining why humans take risks in some situations, and avoid risks in others, is a complex research field. In the present study we proposed an approach in which risk proneness and risk perception affect RT behaviours. On one hand, risk proneness is considered as a general attitude to any type of risk, so that its influence is transversal to all domains. On the other hand, risk perception is understood as a perceptual bias, which may influence RT differently, depending on the domain. The results of this study constitute a foundation upon which to build in this research area and contribute to the increased understanding of human behaviour in risky situations.

## Data Availability Statement

The original contributions presented in the study are included in the article/Supplementary Material, further inquiries can be directed to the corresponding author/s.

## Ethics Statement

The studies involving human participants were reviewed and approved by Ethical Committee of the Polytechnic University of Valencia (ID P4_18_06_19). The patients/participants provided their written informed consent to participate in this study.

## Author Contributions

MA and CJ-R conceived the idea of the manuscript. CJ-R carried out the experiments and wrote the manuscript. JL-J and JM-M analysed the data. IC contributed to the interpretation of the results and supported CJ-R in writing the manuscript. MA supervised the project. All authors contributed to the article and approved the submitted version.

## Conflict of Interest

The authors declare that the research was conducted in the absence of any commercial or financial relationships that could be construed as a potential conflict of interest.
